# Nitrogen-Doped Hierarchical Meso/Microporous Carbon from Bamboo Fungus for Symmetric Supercapacitor Applications

**DOI:** 10.3390/molecules24203677

**Published:** 2019-10-12

**Authors:** Zhanghua Zou, Yu Lei, Yingming Li, Yanhua Zhang, Wei Xiao

**Affiliations:** 1 College of Materials Science and Engineering, Chongqing University of Technology, Chongqing 400054, China; zzh550338@163.com; 2 Research Institute for New Materials Technology, Chongqing University of Arts and Sciences, Yongchuan, Chongqing 402160, Chinaliyingming130@163.com (Y.L.); zyhcoco@163.com (Y.Z.)

**Keywords:** bamboo fungus, porous carbon, symmetric supercapacitor, alkaline activation, nitrogen doping

## Abstract

We report the synthesis of nitrogen-doped hierarchical meso/microporous carbon using renewable biomass bamboo fungus as precursor via two-step pyrolysis processes. It is found that the developed porous carbon (NHPC-800) features honeycomb-like cellular framework with well-developed porosity, huge specific surface area (1708 m^2^ g^−1^), appropriate nitrogen-doping level (3.2 at.%) and high mesopore percentage (25.5%), which are responsible for its remarkable supercapacitive performances. Electrochemical tests suggest that the NHPC-800 electrode offers the largest specific capacitance of 228 F g^−1^, asplendid rate capability and stable electrochemical behaviors in a traditional three-electrode system. Additionally, asymmetric supercapacitor device is built based on this product as well. An individual as-assembled supercapacitor of NHPC-800//NHPC-800 delivers the maximum energy density of 4.3 Wh kg^−1^; retains the majority of capacitanceat large current densities; and shows terrific cycling durability with negligible capacitance drop after long-term charge/discharge for beyond 10,000 cycles even at a high current density of 10 A g^−1^. These excellent supercapacitive properties of NHPC-800 in both three- and two-electrode setups outperform those of lots of biomass-derived porous carbons and thus make it a perspective candidate for producing cost-effective and high-performance supercapacitors

## 1. Introduction

To meet the growing demand for hybrid electric vehicles, flexible electronics and portable power tools, porous carbon-based supercapacitors, which are typical electrical double-layer capacitors (EDLCs), have attracted considerable interest due to the merits of high power densities and superior cycling stability as compared to secondary batteries [1,2,3]. Particularly, porous carbons derived from natural substances stand out, since the renewable nature, easy fabrication process along with abundant availability of precursors could make them quite suitable for production and application on a large scale. As a matter of fact, lots of biomass, including bagasse, wheat straws, Sichuan pepper, pig nails, egg white, corn cob and microorganism were adopted as raw materials to prepare porous carbons by means of some well-established strategies like template synthesis and various activation methods [4,5,6,7,8,9,10,11,12,13]. The resulting biomass-derived porous carbons commonly possess plentiful micropores and high specific surface area but lack sufficient mesopores and macropores. Unfortunately, most of the microporesare not accessible to electrolyte ions on account of their narrow width of <2 nm, resulting in limited specific capacitance, poor rate performances and low energy densities of the corresponding supercapacitors. It has been proven that mesopores with the pore size range from 2 to 50 nm can not only provide effective accessible surface area but also act as spacious channel for quick penetration of electrolyte ions to micropores, while macropores (whose pore width >50 nm) are helpful to shorten the diffusion distance of electrolyte ions into inner pores by offering ion-buffering reservoirs [6,14,15,16]. Accordingly, multilevel interconnected pores (i.e., containing micropores, mesopores and macropores simultaneously) are necessary to guarantee high electrochemical performances of porous carbon-based supercapacitors [3,6,16]. 

Apart from porous texture, the chemical compositions of porous carbons significantly influence their electrochemical properties as well [7,17,18,19,20,21,22]. For instance, introduction of heteroatoms like N, P and S into porous carbons has been proven to be an effective approach to improve their electrochemical behaviors [7,17,18,19,20,21,22]. Especially for nitrogen doping, the electronegative difference between C and N atoms brings about polarized interface, thus elevating the hydrophilicity of porous carbons [21,22]. Besides, the nitrogen-containing functional groups can release extra pseudo capacitance through participation of electrochemical redox reactions, which also contributes to the overall capacitance [19,20,21,22]. Even so, lots of nitrogen-doped porous carbon electrodes still suffer from limited specific capacitance with poor rate capability owing to their low doping level of nitrogen as well as insufficient accessible surface and pore channel. In this context, to upgrade the supercapacitive performances of porous biomass carbons, choosing appropriate precursors to construct hierarchical porous architecture with high nitrogen content is regarded as an ideal design but remains a challenging task.

**Figure 1:**
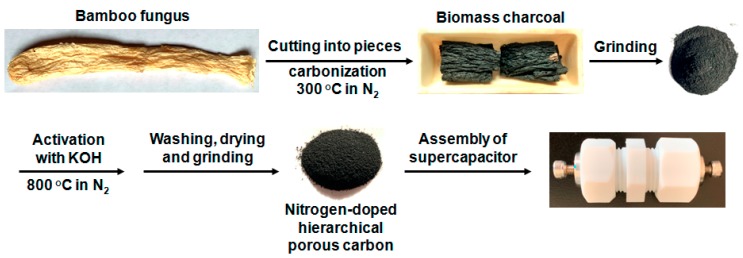
Schematic illustration of fabrication of bamboo fungus-derived nitrogen-doped hierarchical meso/microporous carbon for supercapacitor application.

Bamboo fungus is a kind of cryptogam fungi, which parasitizes in the roots of withered bamboo and has high protein content of over 20 wt.% [23]. Normally, it is regarded as table delicacies and can be used for some medicinal purposes such as the reduction of blood pressure and cholesterol [23]. In spite of these merits, expanding more applications of this biomass into other aspects like energy storage seems to be significant as well. Considering its abundance of nitrogen element, herein, bamboo fungus is utilized as a green and sustainable source to synthesize nitrogen-doped hierarchical porous carbon (NHPC) through carbonization and alkaline activation processes (Figure 1). Benefiting from the three-dimensional meso/microporous texture, well-developed porosity, large specific surface area and proper nitrogen doping level, the currently fabricated nitrogen-doped porous biomass carbon showed impressive supercapacitive properties. For instance, the highest specific capacitance of 228 F g^−1^ and the maximum energy density of 4.3 Wh kg^−1^ are fulfilled in a three-electrode setup andin an individual two-electrode symmetric supercapacitor device with decent rate performance and long-term durability, respectively. As a consequence, this study not only presents a low-cost and perspective porous carbon for supercapacitor applications, but also provides a new use of bamboo fungus.

## 2. Results and Discussion

### 2.1. Characterizations of Bamboo Fungus-Derived NHPC-800 

**Figure 2:**
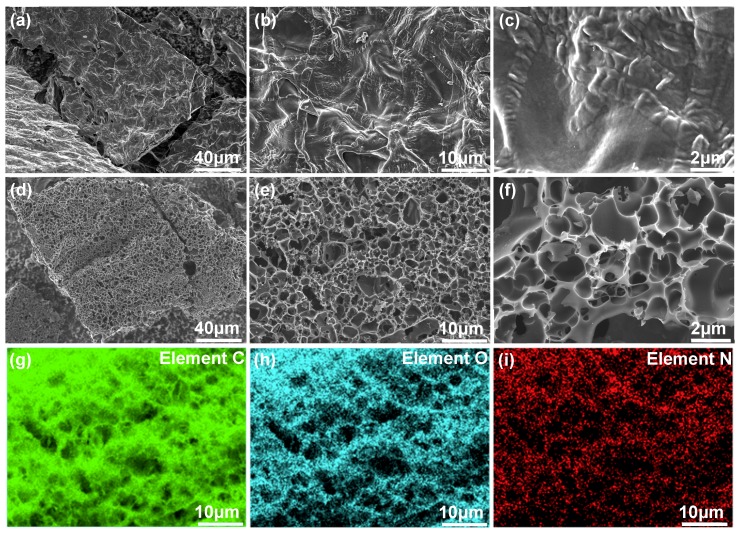
FESEM images of (**a**–**c**) contrastive sample CC and (**d**–**f**) NHPC-800 porous carbon at different magnifications. (**g**,**h**) EDS mapping images of NHPC-800 for the observation domain shown in (**i**), which are assigned to elements C, O and N, respectively.

**Figure 3:**
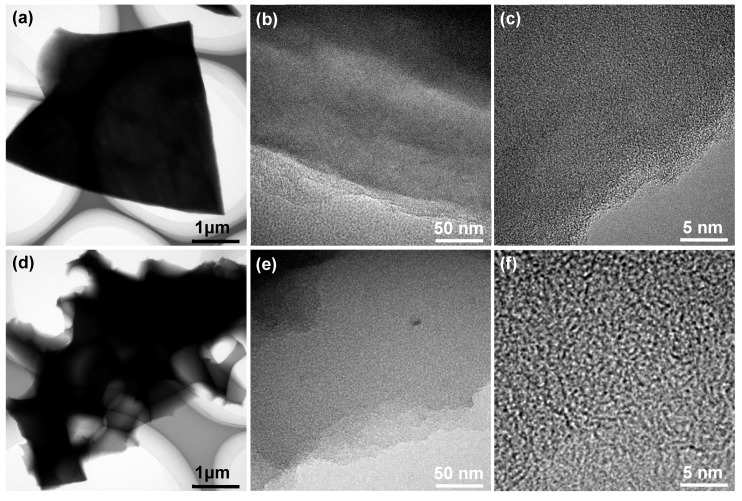
TEM images of (**a**–**c**) contrastive sample CC and (**d**–**f**) NHPC-800 porous carbon at varied magnifications.

Figure 2a–c are field emission scanning electron microscopy (FESEM) images of the sample of contrastive carbon (CC), which is fabricated without potassium hydroxide (KOH) activation. As can be seen, nonporous texture is identified in this carbon material, and such microscopic morphology is not beneficial for the adsorption/desorption and diffusion of electrolyte ions when it is utilized as active electrode material. By contrast, substantial pores with interconnected cellular network and thin pore walls are created in NHPC-800 (Figure 2d–f), which should derive from the alkaline activation at high temperature. The elemental distribution of NHPC-800 is also analyzed by energy-dispersive spectroscopy (EDS). As expected, elements of C, O and N are detected and well distributed in the monitored area of the specimen (Figure 2g,h), indicating the successful doping of N atoms in this product. The transmission electron microscopy (TEM) images of CC and NHPC-800 are presented in Figure 3. Obviously, the former (Figure 3a–c) still shows solid characteristics, while the later (Figure 3d,e) features hierarchical porous structure with a large number of internal micropores throughout the observation scope. These microscopic inspections confirm the well-developed porosity of currently synthesized NPHC-800.

**Figure 4:**
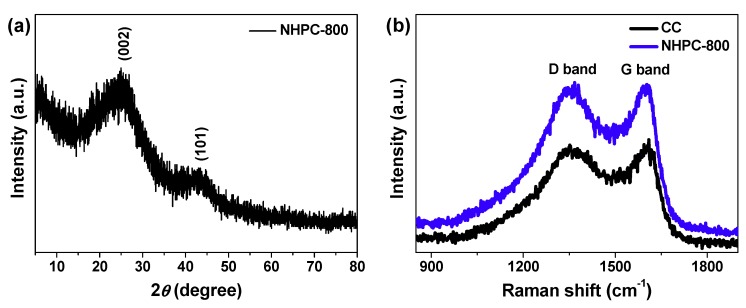
(**a**) XRD pattern of NHPC-800; (**b**) Raman spectra of CC and NHPC-800.

Figure 4a is the X-ray diffraction (XRD) pattern of NHPC-800, where two peaks centered at 2θ = 25.1° and 43.8° are visible, corresponding to its (002) and (101) crystalline planes, respectively [3,5,6]. The broad width and low intensity of the diffraction peaks manifest an amorphous structure [5,14]. Figure 4b depicts Raman spectra of NHPC-800 and CC. There are a couple of bands in both Raman curves, which should be the D (located around 1350 cm^−1^) and G (located around 1600 cm^−1^) bands [3,6,7]. Generally, D band comes from the textural defect and disorder, and G band is associated with the in-plane stretching mode vibration [3,6,7]. It is known that the ordered degree of carbon materials can be evaluated by their intensity ratio of D to G band (I_D_/I_G_). That is, the lower value of I_D_/I_G_ usually means higher ordered structure [3,5,6]. The I_D_/I_G_ value of NHPC-800 (1.02) is clearly larger than that of CC (0.95), suggesting highly disordered structure of NHPC-800, which should be attributable to the numerous pores arising from KOH etching [3,16].

**Figure 5:**
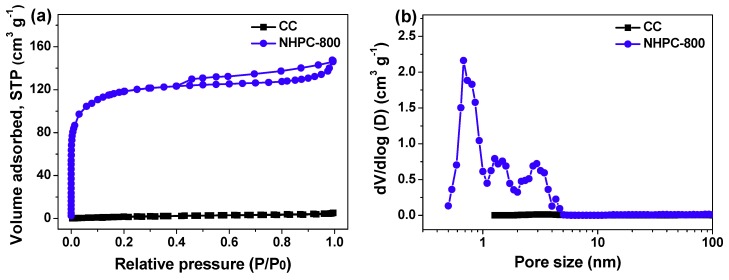
(**a**) Nitrogen adsorption/desorption isotherm curves of CC and NHPC-800; (**b**) Diagrams of their pore size distribution.

N_2_ adsorption/desorption experiment is a facile methodology to investigate porous nature of carbon materials. Based on this test, the isotherm curves of CC and NHPC-800 are given in Figure 5a. Nonporous structure of contrastive sample CC is further confirmed, since its isotherm curve seems to be a horizontal line with quite low adsorbed volume in the entire relative pressure range [6,7]. Differing from CC, NHPC-800 has a combined type I/IV isotherm curve with a typical H3 hysteresis loop [14,24,25]. The dramatic uptake of nitrogen at relative pressure below 0.01 is indicative of substantial micropores [14,24,25]. The hysteresis loop at relative pressure between 0.4 and 1.0 results from capillary condensation taking place in mesopores [14,23]. These findings reflect that NHPC-800 is rich in micropores and mesopores, and such hierarchical meso/microporous texture is intuitively disclosed by its corresponding diagram of pore size distribution (Figure 5b). Porosity parameters of the two specimens are calculated as well. The CC has specific surface area and total pore volume of only 8.6 m^2^ g^−1^ and 0.008 cm^3^ g^−1^, while those of NHPC-800 reach up to 1708 m^2^ g^−1^ and 0.71 cm^3^ g^−1^. In addition, its mesopore surface area and mesopore volume are as high as 436 m^2^ g^−1^ and 0.28 cm^3^ g^−1^, demonstrating the excellent hierarchical porous characteristic and well-developed porosity. It is assumed that the large pore volume and huge specific surface area with suitable distribution of mesopores and micropores may favor the transportation and electrostatic accumulation of electrolyte ions, therefore significantly contributing to the electrochemical properties of NHPC-800 electrode and corresponding symmetric supercapacitor device [3,6,7,26]

**Figure 6:**
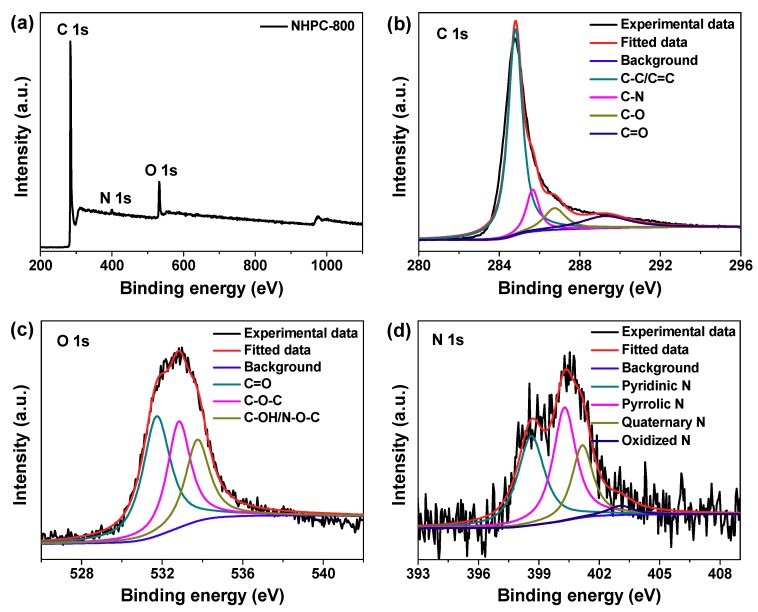
(**a**) X-ray photoelectron spectroscopic (XPS) wide-survey-scan spectrum of synthesized NHPC-800 porous carbon; (**b**–**d**) High-resolution XPS spectra of NHPC-800 porous carbon for the regions of C 1s, O 1s and N 1s, respectively.

The surface element composition and chemical state of NHPC-800 was analyzed by XPS. Three typical peaks are noticed at approximately 284.8, 400.1 and 531.9 eV in the full-survey-scan spectrum (Figure 6a), which should be indexed to the signals of C 1s, N 1s and O 1s, respectively, further verifying the incorporation of nitrogen in this product. The atomic percentage of doped nitrogen within NHPC-800 is calculated to be 3.2%, and it may enhance the overall capacitance via contribution of some pseduo capacitance during charge/discharge process. The high-resolution XPS spectra of the detected three elements are collected as well. For C 1s peak shown in Figure 6b, it is fitted into four parts centered at 284.8, 285.7, 286.7 and 289.3 eV, corresponding to C−C/C=C, C−N, C−O and C=O bonding, respectively [27,28,29,30]. For O 1s region exhibited in Figure 6c, several oxygen-containing groups including C=O (531.6 eV), C−O−C (532.8 eV) and C−OH/N−O−C (533.8 eV) bonding are apparently demonstrated [14,27,31,32]. Besides, the deconvolution of N 1s spectrum reveals four types of nitrogen-containing groups doped in carbon matrix (Figure 6d), which should belong to pyridinic, pyrrolic, quaternary and oxidized N with thefitted peaks located at 398.6, 400.2, 401.2 and 403.14 eV, respectively [7,14,27,33]. As documented in previous reports, the presence of pyridinic and pyrrolic N atoms would increase wettability of porous carbons, take part in electrochemical redox reactions, and thus improve the supercapacitive behaviors [7,14,27,33].

### 2.2. Electrochemical Properties

**Figure 7:**
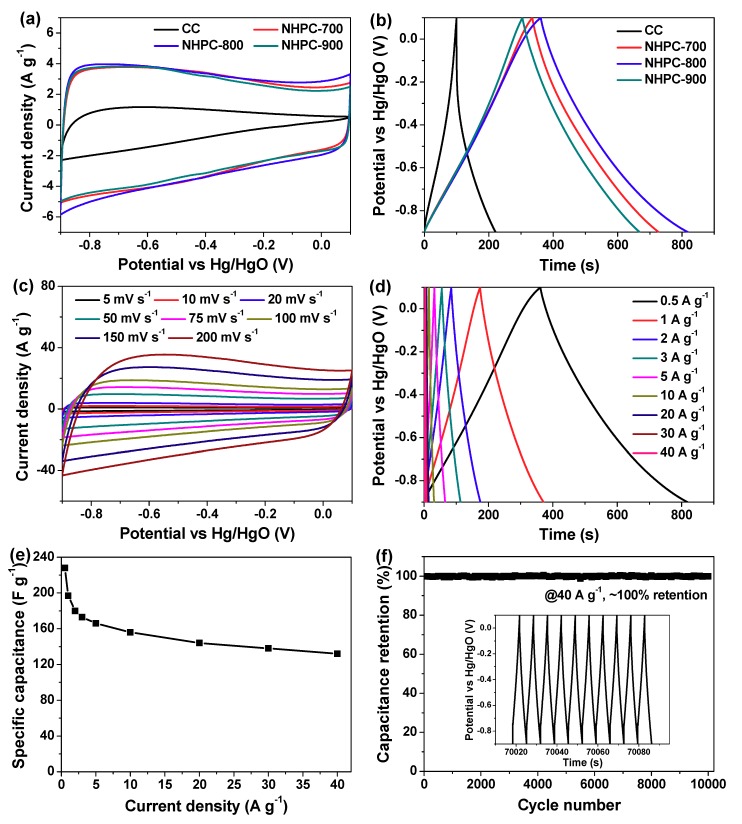
(**a**) CV curves at 20 mV s^−1^ and (**b**) GCD curves at 0.5 A g^−1^ for theas-fabricated CC, NHPC-700, NHPC-800 and NHPC-900 electrodes; (**c**) CV curves obtained at a set of sweep rates and (**d**) GCD curves recorded at a group of current densities for the optimal NHPC-800 electrode; (**e**) Plot of the C_s_ values of NHPC-800 electrode against the applied current densities; (**f**) Cyclic performances of this NHPC-800 electrode.

The three-electrode system was first employed to evaluate the electrochemical performances of currently developed carbon materials by adopting aqueous electrolyte of 2 M KOH. Their cyclic voltammetry (CV) curves at the same scan rate of 20 mV s^−1^ are shown in Figure 7a. Except for contrastive CC electrode, all the NHPC-700, NHPC-800 and NHPC-900 electrodes display quasi-rectangular CV curves, manifesting the dominant EDLC characteristics [6,7,27]. Meanwhile, a slight distortion with wide Faradaic humps located in the potential range from −0.9 V to −0.1 V (vs Hg/HgO) is also available, which should be caused by the electrochemical redox reactions of nitrogen- and oxygen-based moieties [14,33]. Namely, the main EDLC capacitance and part of pseudo capacitance coexist in these nitrogen-doped carbon electrodes and contribute to charge storage together [14,33]. Additionally, it can be seen that the current response and enveloped area of CV curve of NHPC-800 electrode is the highest, implying the largest specific capacitance [7,27,33]. Similar information can be obtained by comparing the corresponding galvanostatic charge/discharge (GCD) curves at the identical current density of 0.5 A g^−1^, since NHPC-800 electrode achieves the longest discharge time as well (Figure 7b). Therefore, a series of CV and GCD measurements of NHPC-800 electrode were further conducted. As exhibited in Figure 7c,d, all the CV curves retain quasi-rectangular shapes and all the GCD curves are nearly isosceles triangles, demonstrating the ideal supercapacitive behaviors and commendable reversibility of NHPC-800 electrode [5,6,33,34]. The specific capacitance (C_s_) of NHPC-800 electrode is deduced in terms of Equation (1) based on these collected GCD curves, which is profiled in Figure 7e. It is clear that the C_s_ value gradually declines as the current density ascends, and the maximum one is as high as 228 F g^−1^ at a low current density of 0.5 A g^−1^. Of note, this C_s_ value is not only preferable to that of NHPC-700 (196 F g^−1^), NHPC-900 (183 F g^−1^) and CC (62 F g^−1^) electrodes but also better than the largest specific capacitance of a number of biomass-derived porous carbon electrodes [29,33,35,36,37,38,39,40,41]. When high current densities of 10 and 40 A g^−1^ are applied, the C_s_ values still reach up to 156 and 132 F g^−1^, respectively. As such, the rate capability of NHPC-800 electrode is desirable. To evaluate the cyclic performance of NHPC-800 electrode, repetitive GCD test was carried out. Although it undergoes 10,000-cycle charge/discharge at current density of 40 A g^−1^, there is negligible capacitance fading throughout this measurement (Figure 7f), and the GCD curve for the last ten cycles seems to be intact (inset of Figure 7f), testifying the splendid electrochemical stability of NHPC-800 electrode.

**Figure 8:**
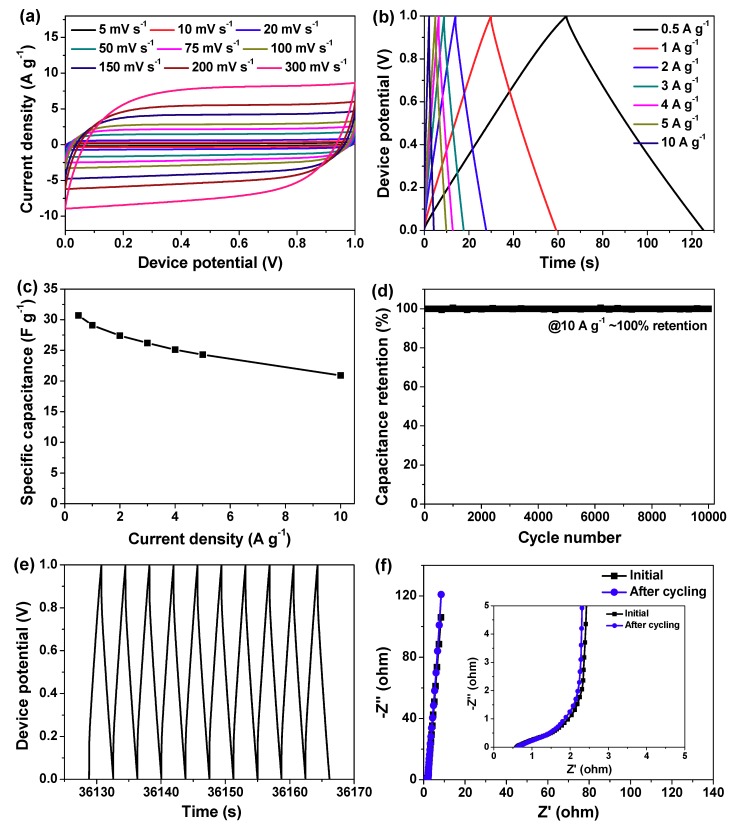
Electrochemical performances of a single as-prepared symmetric supercapacitor device of NHPC-800//NHPC-800. (**a**) CV curves at different sweep rates; (**b**) GCD curves at varied current densities; (**c**) Plot of C_device_ versuscurrent density; (**d**) Cyclic performance of our developed device tested through consecutive GCD measurement at a large current density of 10 A g^−1^ for over 10,000 cycles; (**e**) GCD curves of this cycling stability experiment for the last ten cycles; (**f**) The initial and post-cycling Nyquist plots, and the enlarged view of their high-frequency region is shown in the inset.

Furthermore, we established a symmetric supercapacitor device (i.e., NHPC-800//NHPC-800) by using two identical NHPC-800 electrodes, and its supercapacitive properties were systematically studied in 2 M of KOH solution. Figure 8a gives a set of CV curves at sweep rates of 5−300 mV s^−1^. As envisioned, all of them are close to rectangular shape without significant deformation. The results of GCD tests at the current densities of 0.5−10 A g^−1^ are exhibited in Figure 8b, where quasi-isosceles triangular shape is available with small potential drop, commendable linearity as well as excellent mirrored symmetry between charge curves and their discharge counterparts.

These findings illustrate that the currently developed NHPC-800//NHPC-800 supercapacitor device possesses low internal resistance and high reversibility with rapid charge/discharge rates [5,6,7,16]. Based on Equation (2), the largest specific capacitance of this device (C_device_) is deduced to be 30.6 F g^−1^, and the variation of C_device_ value against current density are plotted in Figure 8c. Compared with the maximum one, C_device_ value attenuates to 20.9 F g^−1^ by boosting current density from 0.5 to 10 A g^−1^. That is, capacitance retention of up to 68.1% is realized at such a large current density, which means the prominent rate capability of our device. Figure 8d presents the cyclic performance of NHPC-800//NHPC-800 supercapacit or device based on consecutive GCD test for more than 10,000 cycles. From beginning to end, capacitance decay can be almost ignored, and the GCD curve for the final ten cycles remains good enough (Figure 8e). Moreover, electrochemical impedance spectroscopy (EIS) measurements show that the Nyquist plots of the device before and after such long-term durability measurement are approximately overlapped (Figure 8f), once again confirming its exceptional cycling stability.

**Figure 9:**
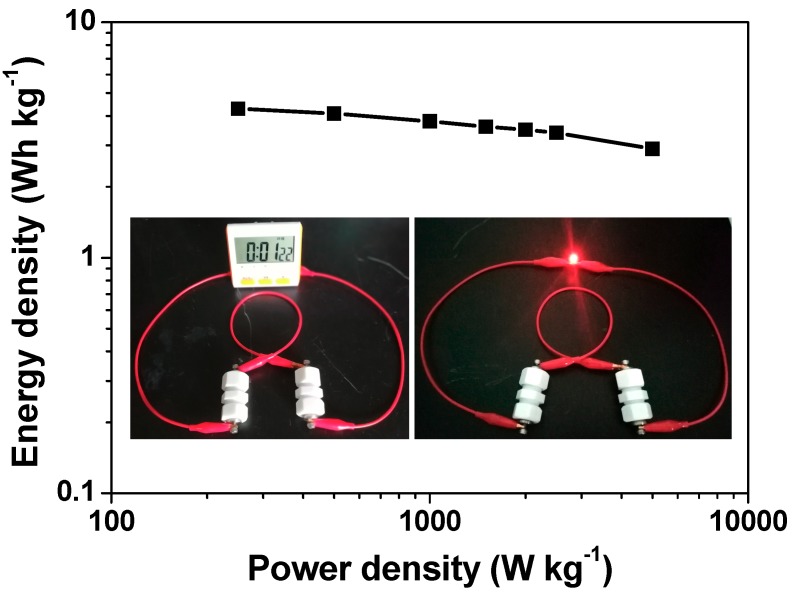
Ragone plot of an individual symmetric supercapacitor device of NHPC-800//NHPC-800; the insets presents the optical pictures of a commercial timer and a red LED bead well driven by two NHPC-800//NHPC-800 supercapacitor cells connected in series.

According to Equations (3) and (4), the energy densities of single NHPC-800//NHPC-800 supercapacitor device are calculated, which are 4.3, 4.1, 3.8, 3.6, 3.5, 3.4 and 2.8 Wh kg^−1^ at power densities of 0.25, 0.5, 1, 1.5, 2, 2.5 and 5 kW kg^−1^, respectively. The acquired data are also profiled in Figure 9 for intuitive visualization. Interestingly, the largest energy density delivered by currently developed supercapacitor device (4.3 Wh kg^−1^) can be comparable or even superior to that of lots of commercial and biomass porous carbon-based symmetric supercapacitors [6,7,37,42,43,44,45]. Finally, to examine the viability and usefulness of NHPC-800//NHPC-800 supercapacitor, we connect two cells in series to attempt to power some usual electronic products. As a demonstration, such assembled tandem device with total loading amount of 7.8 mg of active material well drives a timer and a red LED bead (whose rated voltages are 1.5 and 2.0 V, respectively) at full charging state, and the LED bulb can even stay bright for beyond 2 min, hence exhibiting the excellent application potential.

## 3. Materials and Methods 

### 3.1. Reagents

Bamboo fungus was purchased from local supermarket. Nickel foam, KOH, HCl, absolute ethanol, polyvinylidene fluoride, *N*-methyl-2-pyrrolidone and acetylene black were provided by Damao Reagent Co., Ltd. (Tianjin, China) and used without further purification.

### 3.2. Synthesis of Bamboo Fungus-Derived NHPC

Two grams of bamboo fungus was cut into small pieces, and then subjected to direct pyrolysis at 300 °C for 2 h in quartz tube furnace under N_2_ flow. Next, 0.6 g of the resulting biomass charcoal was ground into fine powder, moistened with 1 mL of ethanol and successively mixed with 0.6 g of KOH dissolved in 1 mL of water to form a slurry, which was completely dried at 120 °C. For further creation of porosity, the resulting solid mixture was gathered in acorundum boat and activated in quartz tube furnace at different temperature (700, 800 and 900 °C, ramp rate: 2 °C min^−1^) for 2 h under N_2_ atmosphere. At last, the obtained crude products were sufficiently washed by 2 M of HCl and abundant water to remove impurities, followed by drying and grinding, hence producing nitrogen-doped hierarchical porous carbons. In the present study, they are denoted as NHPC-T for convenience, where T means the above activation temperature. For comparison, contrastive carbon material was also prepared by directly pyrolyzing the biomass charcoal at 800 °C in the absence of KOH, which was denoted as CC.

### 3.3. Characterizations

The morphologies of the specimens were examined by field emission scanning electron microscopy (FESEM, Zeiss Gemini SEM 300) and transmission electron microscopy (TEM, JEOL JEM-2100F) working at acceleration voltages of 3 and 200 kV, respectively. X-ray diffraction (XRD) measurement was done on powder diffractometer (Tongda TD-3500) with radiation source of monochromatic Cu Kα (λ = 0.154156 nm). Raman spectra were collected on Raman spectrometer (Zolix Finder One) adopting 532 nm laser source. X-ray photoelectron spectroscopic (XPS) spectra were recorded on VG ESCALAB MARK II instrument utilizing focused Mg Kα X-ray source (hν = 1253.6 eV). Porosity properties of the samples were characterized by N_2_ adsorption/desorption experiments, which were carried out on Micromeritics ASAP2020 analyzer at 77 K.

### 3.4. Electrochemical Measurements and Corresponding Calculations

The working electrodes were fabricated as follows. In brief, active material (NHPC-T), polyvinylidene fluoride binder and acetylene black were mixed together at mass ratio of 75:10:15, followed by dispersion in a little of *N*-methyl-2-pyrrolidone to yield a homogeneous slurry. Afterwards, it was uniformly pressed onto nickel foam under 4 MPa and then sufficiently dried for further use. The area of the zone coated by such black slurry on each electrode was ~1 cm^2^, which contained ~2 mg of active material.

All the electrochemical tests were conducted on a CHI 760E electrochemical workstation. For the measurements in three-electrode setup, aqueous solution of 2 M KOH was adopted as the electrolyte and rectangular nickel foam loaded with NHPC-T samples, Hg/HgO electrode as well as platinum foil were used as the working, reference and counter electrodes, respectively. The specific capacitance of a single electrode was deduced from galvanostatic charge/discharge (GCD) curves by the following Equation (1),

C_s_ = It/ΔVm (1)

where C_s_ (F g^−1^), I(A), t (s), ΔV(V) and m(g) refer to specific capacitance of working electrode, discharge current, discharge time, discharge potential range and loading weight of active material, respectively [6,7,14,16].

Moreover, a symmetric supercapacitor device was built as well. Briefly, two pieces of NHPC-800-loaded circular nickel foam were paired together with a hydrophilic thin cellulose film sandwiched in between and thereafter sealed in a two-electrode setup, where appropriate amount of 2 M KOH was added as electrolyte in advance. Electrochemical properties of the assembled supercapacitor device, including CV and GCD curves, were systematically determined. EIS analysis of the supercapacitor device was surveyed at open circuit with signal amplitude of 5 mV. The specific capacitance, energy and power densities of the supercapacitor device were computed from its GCD curves by the following three equations,

C_device_ = It/ΔVm (2)

E = (C_device_ΔV^2^)/7.2 (3)

P = 3600E/t(4)

where C_device_ (F g^−1^), I(A), t (s), ΔV(V), m(g), E (Wh kg^−1^) and P (W kg^−1^) stand for specific capacitance of the supercapacitor device, discharge current, discharge time, output potential difference, total mass of NHPC-800porous carbon within the device, energy density and power density, respectively [3,6,7,16].

## 4. Conclusions

A nitrogen-doped hierarchical meso/microporous carbon (i.e., NHPC-800) was developed by direct pyrolysis of bamboo fungus, followed by high-temperature alkaline activation. The synthesized NHPC-800 porous carbon features appropriate nitrogen content of 3.2 at.% and well-developed porosity with huge specific surface area of 1708 m^2^ g^−1^ as well as rational distribution of mesopores and micropores. The textural and porous characteristics endow NHPC-800 with impressive supercapacitive properties. When evaluated in a conventional three-electrode setup, the NHPC-800 electrode releases high specific capacitance (228 F g^−1^ at 0.5 A g^−1^), has outstanding rate performance (132 F g^−1^ at 40 A g^−1^) and displays terrific electrochemical durability. Later, a symmetric supercapacitor device is constructed by using NHPC-800 porous carbon as active material, which offers an impressive energy density of 4.3 Wh kg^−1^, features excellent rate capability, and achieves negligible capacitance degradation after extensive charge/discharge. These findings imply that our nitrogen-doped hierarchical meso/microporous carbon fabricated from bamboo fungus may be a promising candidate toward realistic applications in an advanced energy storage area.
